# Association of statin use on survival outcomes of patients with early-stage HER2-positive breast cancer in the APHINITY trial

**DOI:** 10.1007/s10549-025-07699-2

**Published:** 2025-04-28

**Authors:** Christian Maurer, Elisa Agostinetto, Lieveke Ameye, Matteo Lambertini, Samuel Martel, Noam Ponde, Mariana Brandão, Francesca Poggio, Arlindo Ferreira, Rachel Schiff, Carmine De Angelis, Richard D. Gelber, Susan Dent, Christoph Thomssen, Martine Piccart, Evandro de Azambuja

**Affiliations:** 1 National Center for Tumor Diseases (NCT) Heidelberg, University Hospital and German Cancer Research Center Heidelberg, Im Neuenheimer Feld 460, 69120 Heidelberg, Germany; 2 Department I of Internal Medicine, Center for Integrated Oncology Aachen Bonn Cologne Düsseldorf, Faculty of Medicine and University Hospital Cologne, University of Cologne, Cologne, Germany; 3 Université Libre de Bruxelles (ULB), Hôpital Universitaire de Bruxelles (HUB), Institut Jules Bordet, Rue Meylemeersch 90, 1070 Brussels, Belgium; 4 Department of Internal Medicine and Medical Specialties (DiMI), School of Medicine, University of Genova, Genoa, Italy; 5 Department of Medical Oncology, U.O. Clinical Di Oncologia Medica, IRCCS Ospedale Policlinico San Martino, Genoa, Italy; 6 Specialised Medicine Department, CISSS Montérégie-Centre/Hôpital Charles-Le Moyne, Greenfield Park, Québec, Canada; 7 Université of Sherbrooke, Sherbrooke, Québec Canada; 8 Clinical Development Department, Daiichi Sankyo, Morristown, NJ USA; 9 Católica Medical School, Universidade Católica Portuguesa, Lisbon, Portugal; 10 Lester and Sue Smith Breast Center, Departments of Medicine and of Molecular and Cellular Biology, Baylor College of Medicine, Houston, TX USA; 11 Medical Oncology Unit, Department of Clinical Medicine and Surgery, University of Naples “Federico II”, Naples, Italy; 12 Harvard Medical School, Harvard TH Chan School of Public Health, Dana-Farber Cancer Institute, Frontier Science Foundation, Boston, MA USA; 13 Wilmot Cancer Institute, Department of Medicine, University of Rochester, Rochester, NY USA; 14 Department of Gynaecology, Martin-Luther-University Halle-Wittenberg, Halle (Saale), Germany

**Keywords:** Breast cancer, HER2-positive, Statins, Pertuzumab, Trastuzumab

## Abstract

**Purpose:**

There is evidence that statins might improve the outcome of patients with breast cancer. The role of statins in patients with early HER2-positive breast cancer is unknown. Therefore, we explored the association between statin use and survival outcomes in early HER2-positive breast cancer patients in the phase III APHINITY trial (adjuvant pertuzumab/trastuzumab).

**Methods:**

All patients (intent-to-treat population, *n* = 4804) were included (6.2 years median follow-up database). The primary objective was to investigate the association of statin use on invasive disease-free survival (IDFS), distant relapse-free interval (DRFI), and overall survival (OS). Patients who received statins at baseline, or started statins within 1 year from randomization were considered statin users. Survival curves were estimated using the Kaplan–Meier method. We used a Cox proportional hazards model for multivariate analysis.

**Results:**

Overall, 423 (8.8%) patients were classified as statin users. They were older, more often postmenopausal, had a higher body mass index, more often diabetes, hypertension, coronary heart disease and hyperlipidemia, had smaller sized tumors, were treated more often with breast conserving surgery, and less often with anthracycline-containing regimens. Overall, 508 IDFS events (12.8% among statin users and 10.4% among non-statin users) and 272 deaths (8.5% and 5.4%, respectively) occurred. In multivariate analysis, statin use was not associated with IDFS (HR, 1.11; 95% CI, 0.80–1.52), DRFI (HR, 1.21; 95% CI, 0.81–1.81) nor OS (HR, 1.16; 95% CI, 0.78–1.73).

**Conclusion:**

In APHINITY, statin use was not associated with improved survival outcomes. These results must be interpreted with caution due to the exploratory nature of the analysis and the associated limitations.

**Supplementary Information:**

The online version contains supplementary material available at 10.1007/s10549-025-07699-2.

## Introduction

About 15%−20% of all breast cancers (BC) show overexpression/amplification of the human epidermal growth factor receptor 2 (HER2). In the absence of specific anti-HER2 treatments, HER2-positive (HER2+) BC is an aggressive BC subtype with worse prognosis than hormone receptor-positive disease. Targeting the HER2 pathway has led to marked improvement in the outcome of patients in both early and advanced disease settings [1, 2]. However, despite the efficacy of anti-HER2 drugs and the improved outcomes seen with combination of different HER2-pathway inhibitors, resistance remains a clinical challenge [3].

Comorbidities are present in 32–42% of BC patients, with hypertension being most common, followed by other cardiovascular diseases and type 2 diabetes [4]. Accordingly, many patients with BC are prescribed medications, including statins, for such conditions. In addition to cholesterol biosynthesis, the mevalonate pathway, via isoprenoid intermediates, can generate cell proliferative and survival signals [5]. Preclinical evidence suggested that the mevalonate pathway might play a role in tumor initiation and progression [6]. The rate-limiting enzyme of the mevalonate pathway is the hydroxymethylglutaryl coenzyme A reductase, which can be inhibited by statins. Preclinical work, including BC models, suggested that statins might induce apoptosis and reduce tumor growth, angiogenesis, and metastases [7, 8]. Observational studies have provided conflicting data regarding the possible impact of statins on breast cancer outcomes. The definition of statin users differed between the studies which makes comparability difficult. Some observational studies, including two meta-analyses, suggested a protective association between statin use and breast-cancer specific death and/or overall survival (OS) [9–22], while other studies have not shown a positive effect of statins on this survival outcome [23–31]. Regarding BC recurrence, some studies have reported that statin use correlates with better outcomes [16–18, 21, 25, 29, 32–35], while others have not found this association [20, 27, 28, 30, 36–39].

Preclinical models suggested that the mevalonate pathway acts as an escape mechanism of survival and growth in HER2 + BC resistant to anti-HER2 therapies. Inhibitors of the pathway with simvastatin resulted in apoptosis and growth inhibition of resistant BC cells [40]. Examining the association between statin use and the outcome of patients with HER2 + BC is therefore warranted. Accordingly, we investigated the association between statin use and survival outcomes in patients with early HER2 + BC enrolled in the APHINITY trial.

## Methods

APHINITY is a prospective, randomized, double-blind phase 3 trial that tested the addition of pertuzumab to trastuzumab and chemotherapy as adjuvant treatment for patients with early HER2 + BC. Overall, 4805 patients were randomly assigned to receive chemotherapy and trastuzumab plus either pertuzumab or placebo. The addition of pertuzumab was associated with an improved invasive disease-free survival (IDFS). Yet this benefit was restricted to patients with node-positive disease. Detailed information regarding study design, eligibility, conduct and results were previously reported [41–43].

### Study population

All patients in the intent-to-treat population (*n* = 4804) were included in the current analysis, at a median follow-up of 6.2 years. Trial baseline case report forms collected co-medications and investigators were asked to document any new treatment, irrespective of its duration, throughout the conduct of the trial. Patients who received statins at baseline, or started statins within 1 year from randomization were considered statin users. To prevent lead-time-bias, patients with start of statins more than 1 year from randomization were considered non-users.

### Primary outcome

The primary objective of this sub-study was to determine the association between statin use and outcomes in terms of IDFS, distant relapse-free interval (DRFI), and OS. For IDFS, DRFI and OS the same definitions as in the APHINITY trial were used [41]. IDFS is defined as the time from randomization until the date of the first occurrence of ipsilateral invasive breast tumor, recurrence of ipsilateral locoregional invasive disease, a distant disease recurrence, contralateral invasive BC, or death from any cause. DRFI is defined as the time between randomization and the date of distant BC recurrence. OS is defined as the time from randomization to death due to any cause.

### Statistical analysis

Patients’ and tumor baseline characteristics were compared with respect to the presence or absence of statin co-medication using the chi-square test. Survival curves for IDFS, DRFI, and OS according to the presence or absence of statin co-medication were estimated using the Kaplan Meier method. In univariate analysis, the association of statin use (statin use, lipophilic and hydrophilic statins = 3 groups) was assessed, stratified by randomized arm (pertuzumab versus [vs] placebo), menopausal status (premenopausal vs postmenopausal), hormone receptor status (negative vs positive; centrally assessed) and body mass index (BMI; normal [20–24.9 kg/m^2^] vs overweight [25.0–29.9 kg/m^2^] vs obese [≥ 30.0 kg/m^2^]). Considering multiple testing with respective type I error increase, a Bonferroni testing was applied and a *p*-value < 0.00167 (i.e., 0.05/30 [10 groups × 3 tests]) was considered statistically significant. A multivariate analysis using a Cox proportional hazard model was used to assess the association of statin use adjusted for age (< 65 vs ≥ 65 years), menopausal status (premenopausal vs postmenopausal), BMI (< 30 kg/m^2^ vs 30–34.9 kg/m^2^ vs ≥ 35 kg/m^2^), tumor size (< 2 cm vs ≥ 2 < 5 cm vs ≥ 5 cm), nodal status (none vs 1–3 vs ≥ 4 positive lymph nodes), hormone receptor status (negative vs positive), existence of comorbidities (diabetes, hypertension and coronary heart disease; as noted by the investigators), and treatment arm (placebo vs pertuzumab). A fixed time Cox model with starting timepoint of randomization was used as primary analysis. Interaction terms were considered between statin use at any time and treatment arm, hormone receptor status and BMI. A multivariate competing risk analysis for OS was performed considering cause of death other than breast cancer as a competing risk. Two sensitivity analyses for the association of statin use with IDFS, DRFI and OS were performed (Cox model adjusting for the same variables as above): 1) considering a landmark at 12 months (i.e., start at 1 year after randomization), and 2) considering start of statin use as a time-dependent variable. Statistical analysis was conducted using SAS 9.4 software (SAS Institute Inc., Cary, NC, USA).

## Results

### Patient population

Of the 4804 patients of the intent-to-treat population, 423 patients (8.8%) were classified as statin users (*n* = 302 only lipophilic statin users, *n* = 116 only hydrophilic statin users and *n* = 5 lipophilic and hydrophilic statin users). For the majority of statin users (*n* = 230, 54.4%), exact start and stop dates of statin treatment were missing. For 42 statin users (9.9%), start and stop dates of statin treatment were available. For these patients, median time on statins was 5 months (interquartile range [IQR], 2–34 months). For the rest of the statin users, either start date (*n* = 32; 7.6%) or end date (*n* = 119; 28.1%) was missing. The majority of statin users (91.5%) were on statin treatment at baseline and during study treatment. Twenty-five patients (5.9%) started statin treatment after randomization (median time from randomization to treatment start, 2.3 months; IQR, 2 days-6.6 months). Two patients started statin treatment more than 1 year after randomization and were considered non-statin users. Table 1 illustrates the baseline characteristics of the study population. Compared to non-statin users, statin users were older (median age, 62 vs 50 years), more frequently postmenopausal (91.1% vs 47.6%), had a higher BMI (median, 27.3 kg/m^2^ vs 24.4 kg/m^2^), had smaller sized tumors (≤ 1.9 cm, 46.1% vs 39.5%), were treated more often with breast conserving surgery than with mastectomy (54.1% vs 44.9%), were treated less frequently with anthracycline-containing regimens (71.4% vs 78.6%) and were more often diagnosed with diabetes (24.6% vs 3.9%), hypertension (64.1% vs 18.4%), coronary heart disease (4.0% vs 0.6%) and hyperlipidemia (83.0% vs 3.4%).

**Table 1:** Baseline characteristics of the study population

Parameters	Statin users	Non-statin users	*p*-value
	*n* = 423	*n* = 4381	
Age			
Median (IQR), years	62 (57–68)	50 (43–58)	< 0.001
< 65 years, *n* (%)	256 (60.5)	3940 (89.9)	
≥ 65 years, *n* (%)	167 (39.5)	441 (10.1)	
Gender			
Female, *n* (%)	418 (98.8)	4375 (99.9)	0.002
Male, *n* (%)	5 (1.2)	6 (0.1)	
Menopausal status			
Postmenopausal, *n* (%)	380 (91.1)	2082 (47.6)	< 0.001
Premenopausal, *n* (%)	37 (8.9)	2288 (52.4)	
Missing, *n*	1	5	
Men, *n*	5	6	
BMI			
Median (IQR), kg/m^2^	27.3 (24.1–31.5)	24.4 (21.9–28.0)	< 0.001
< 30 kg/m^2^, *n* (%)	280 (66.2)	3647 (83.6)	
≥ 30 kg/m^2^ < 35 kg/m^2^, *n* (%)	93 (22.0)	475 (10.9)	
≥ 35 kg/m2, *n* (%)	50 (11.8)	242 (5.5)	
Missing, *n*		17	
Type of surgery			
Breast conserving, *n* (%)	229 (54.1)	1965 (44.9)	< 0.001
Mastectomy, *n* (%)	194 (45.9)	2413 (55.1)	
Missing, *n*		3	
Histology			
Ductal, *n* (%)	387 (91.5)	3945 (90.0)	0.33
Lobular, *n* (%)	12 (2.8)	112 (2.6)	
Mixed, DCIS, LCIS, *n* (%)	8 (1.9)	101 (2.3)	
Other, *n* (%)	16 (3.8)	223 (5.1)	
Nodal status			
0, *n* (%)	166 (39.2)	1633 (37.3)	0.32
1–3, *n* (%)	159 (37.6)	1648 (37.6)	
≥ 4, *n* (%)	98 (23.2)	1100 (25.1)	
Hormone receptor status			
ER and PR−, *n* (%)	144 (34.0)	1488 (34.0)	0.97
ER and/or PR+, *n* (%)	279 (66.0)	2893 (66.0)	
Tumor grade			
1, *n* (%)	6 (1.5)	89 (2.1)	0.72
2, *n* (%)	134 (33.3)	1395 (33.1)	
3, *n* (%)	262 (65.2)	2735 (64.8)	
Unevaluable/unknown, *n*	21	162	
Tumor size			
0–1.9 cm, *n* (%)	195 (46.1)	1726 (39.5)	0.02
2–4.9 cm, *n* (%)	201 (47.5)	2355 (53.8)	
≥ 5 cm, *n* (%)	27 (6.4)	294 (6.7)	
Unknown, *n*		6	
Randomization arm			
Pertuzumab, *n* (%)	201 (47.5)	2199 (50.2)	0.29
Placebo, *n* (%)	222 (52.5)	2182 (49.8)	
Adjuvant chemotherapy			
Anthracycline, *n* (%)	302 (71.4)	3442 (78.6)	0.001
Non-anthracycline, *n* (%)	121 (28.6)	939 (21.4)	
Adjuvant radiotherapy			
No, *n* (%)	115 (27.2)	1207 (27.6)	0.87
Yes, *n* (%)	308 (72.8)	3174 (72.4)	
Adjuvant ET, * n* = 3172			
No, *n* (%)	50 (17.9)	455 (15.7)	0.35
Yes, *n* (%)	229 (82.1)	2438 (84.3)	
Diabetes			
No, *n* (%)	319 (75.4)	4210 (96.1)	< 0.001
Yes, *n* (%)	104 (24.6)	171 (3.9)	
Hypertension			
No, *n* (%)	152 (35.9)	3575 (81.6)	< 0.001
Yes, *n* (%)	271 (64.1)	806 (18.4)	
Coronary heart disease			
No, *n* (%)	406 (96.0)	4356 (99.4)	< 0.001
Yes, *n* (%)	17 (4.0)	25 (0.6)	
Hyperlipidemia			
No, *n* (%)	72 (17.0)	4230 (96.6)	< 0.001
Yes, *n* (%)	351 (83.0)	151 (3.4)	
Any of the above 4 comorbidities			
No, *n* (%)	16 (3.8)	3400 (77.6)	< 0.001
Yes, *n* (%)	407 (96.2)	981 (22.4)	

*BMI* body mass index, *DCIS* ductal carcinoma in situ, *ER* estrogen receptor, *ET* endocrine therapy, in ER+ and/or PR+ disease, *IQR* interquartile range, *LCIS* lobular carcinoma in situ; nodal status, number of positive lymph nodes, *PR* progesterone receptor

### Survival outcomes

**Fig. 1:**
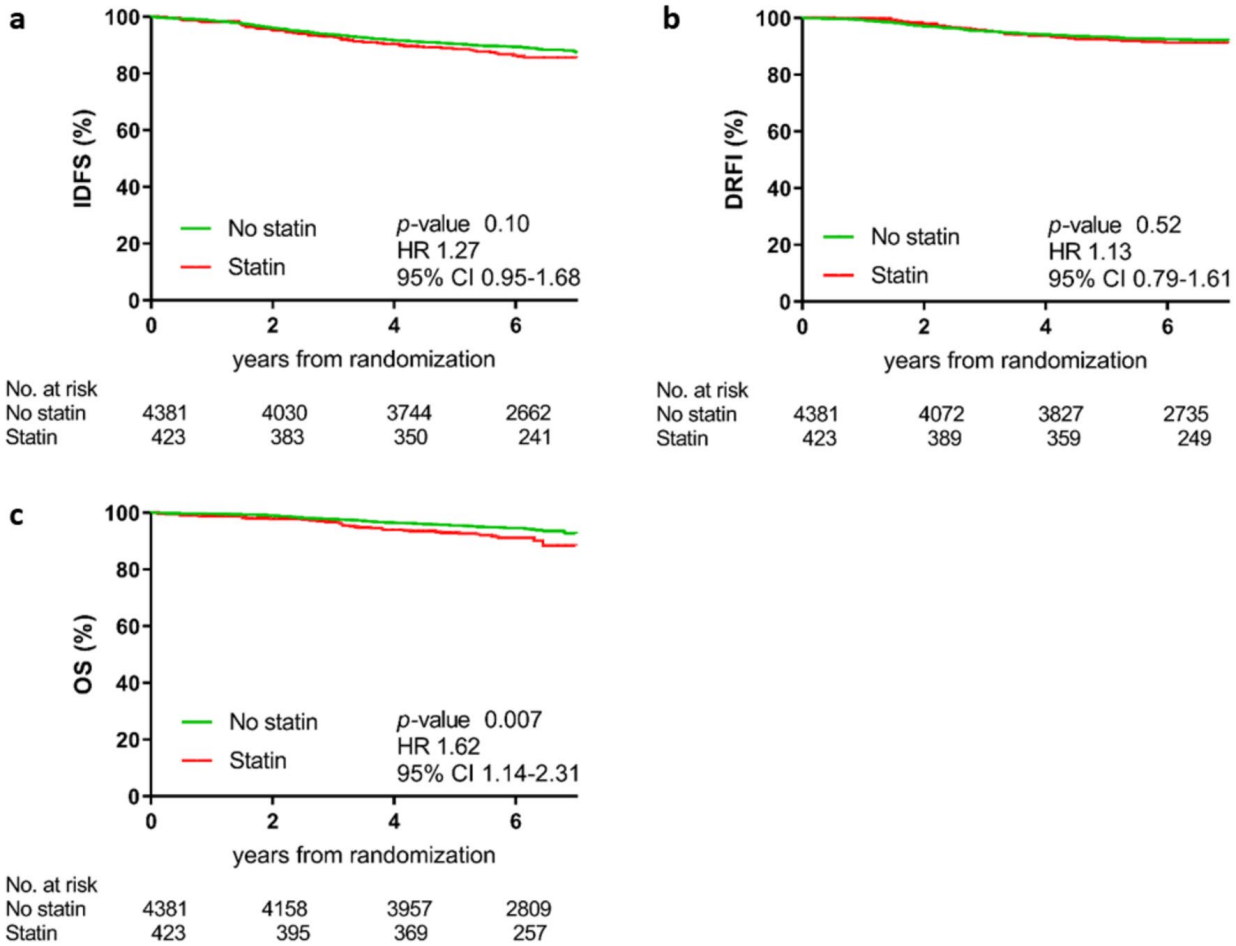
Kaplan Meier curves for survival endpoints unadjusted for patient and disease characteristics. Time, in years, is measured from date of randomization; Twenty-five patients (5.9%) started statin treatment after randomization (median time from randomization to treatment start, 2.3 months; IQR, 2 days–6.6 months). Overall about 95% of statin users were under treatment at randomization (91%) or started within 3 months after randomization (4%). Two patients started statin treatment after 1 year from randomization and were considered non-statin users. Results landmarked from 12 months after study enrolment, and those based on a time-varying Cox model analysis were similar to those depicted in this analysis measured from date of randomization. *CI* confidence interval, *DRFI* distant relapse-free interval, *HR* hazard ratio, *IDFS* invasive disease-free survival, *No.* number, *OS* overall survival

The median follow-up for OS was 73.8 months (IQR, 69.3–75.5 months) for statin users and 74.1 months (IQR, 69.3–75.5 months) for non-statin users. Overall, 508 IDFS events (54/423 [12.8%] among statin users and 454/4381 [10.4%] among non-statin users), 343 DRFI events (33/423 [7.8%] and 310/4381 [7.1%], respectively) and 272 deaths (36/423 [8.5%] and 236/4381 [5.4%], respectively) occurred. The most common cause of death was related to recurrence of BC (*n* = 187; 69%). Of the 36 deaths among statin users, 21 [58.3%] were due to recurrence of disease. Of the 236 deaths among the non-statin users, 166 [70.3%] were due to recurrence of disease. Numerically, there were more non-BC-related deaths in statin users (*n* = 15; 41.7%) than in non-statin users (*n* = 70; 29.7%). Figure 1 shows the Kaplan Meier curves for IDFS, DRFI, and OS. Applying the Bonferroni correction with a *p*-value < 0.00167 for statistical significance, statin use had no association with IDFS (hazard ratio [HR], 1.27; 95% confidence interval [CI], 0.95–1.68; *p*-value = 0.10) or DRFI (HR, 1.13; 95% CI, 0.79–1.61; *p*-value = 0.52) in univariate analysis. Regarding OS, statin use overall was associated with a trend to worse outcome in univariate analysis (HR, 1.62, 95% CI, 1.14–2.31; *p*-value 0.007) without reaching statistical significance.

Supplementary Table 1 (Supplementary Information) shows the univariate analysis of IDFS, DRFI and OS stratified by treatment arm, menopausal status, hormone receptor status and BMI. No subgroup was identified that showed a significant association between statin use and IDFS, DRFI nor OS: When stratified by treatment arm within the APHINITY trial, menopausal status, BMI, tumor size, nodal status or hormone receptor status, treatment with statins in general, lipohilic or hydrophilic statins was not associated with survival outcomes. In multivariate analysis, no association was observed between statin use and IDFS (HR, 1.11; 95% CI, 0.80–1.52; *p*- value = 0.54), DRFI (HR, 1.21; 95% CI, 0.81–1.81; *p*-value = 0.35) or OS (HR, 1.16; 95% CI, 0.78–1.73; *p*-value = 0.45) (Table 2). These findings were confirmed in the two sensitivity analyses (Table 3).

**Table 2:** Multivariate analysis of IDFS, DRFI, and OS

Parameter	Patients, *n*	Events, *n*	Hazard ratio (95% CI)	*p*-value
IDFS				
Statin use				
No	4348	452	1.00	
Yes	417	51	1.11 (0.80–1.52)	0.54
Age group				
< 65 years	4161	429	1.00	
≥ 65 years	604	74	1.30 (0.98–1.72)	0.07
Menopausal status				
Premenopausal	2311	244	1.00	
Postmenopausal	2454	259	0.95 (0.78–1.16)	0.62
Hormone receptor status				
ER and/or PR positive	3148	316	1.00	
ER and PR negative	1617	187	1.10 (0.92–1.32)	0.30
BMI				
< 30 kg/m^2^	3911	404	1.00	
≥ 30 kg/m^2^ < 35 kg/m^2^	563	66	1.09 (0.83–1.42)	0.53
≥ 35 kg/m^2^	291	33	0.94 (0.65–1.36)	0.75
Diabetes				
No	4493	472	1.00	
Yes	272	31	0.93 (0.63–1.37)	0.71
Hypertension				
No	3700	375	1.00	
Yes	1065	128	1.13 (0.90–1.42)	0.30
Coronary heart disease				
No	4724	495	1.00	
Yes	41	8	1.96 (0.96–3.99)	0.06
Nodal status				
0 nodes	1785	94	1.00	
1–3 nodes	1795	176	1.91 (1.48–2.46)	< 0.001
≥ 4 nodes	1185	233	3.86 (3.01–4.95)	< 0.001
Tumor size				
0–1.9 cm	1906	152	1.00	
≥ 2 cm < 5 cm	2541	282	1.20 (0.98–1.47)	0.08
≥ 5 cm	318	69	1.87 (1.39–2.51)	< 0.001
Treatment arm				
Placebo	2383	282	1.00	
Pertuzumab	2382	221	0.78 (0.65–0.93)	0.005
DRFI				
Statin use				
No	4348	309	1.00	
Yes	417	32	1.21 (0.81–1.81)	0.35
Age group				
< 65 years	4161	304	1.00	
≥ 65 years	604	37	0.99 (0.68–1.45)	0.98
Menopausal status				
Premenopausal	2311	179	1.00	
Postmenopausal	2454	162	0.92 (0.72–1.17)	0.48
Hormone receptor status				
ER and/or PR positive	3148	217	1.00	
ER and PR negative	1617	124	1.05 (0.84–1.32)	0.66
BMI				
< 30 kg/m^2^	3911	277	1.00	
≥ 30 kg/m^2^ < 35 kg/m^2^	563	43	1.06 (0.76–1.47)	0.74
≥ 35 kg/m^2^	291	21	0.87 (0.55–1.38)	0.57
Diabetes				
No	4498	324	1.00	
Yes	272	17	0.82 (0.49–1.38)	0.46
Hypertension				
No	3700	265	1.00	
Yes	1065	76	1.01 (0.76–1.35)	0.94
Coronary heart disease				
No	4725	336	1.00	
Yes	41	5	1.85 (0.75–4.56)	0.18
Nodal status				
0 nodes	1785	38	1.00	
1–3 nodes	1795	107	2.75 (1.90–3.99)	< 0.001
≥ 4 nodes	1185	196	7.58 (5.31–10.82)	< 0.001
Tumor size				
0–1.9 cm	1906	87	1.00	
≥ 2 cm < 5 cm	2541	197	1.34 (1.04–1.73)	0.02
≥ 5 cm	318	57	2.19 (1.55–3.09)	< 0.001
Treatment arm				
Placebo	2383	192	1.00	
Pertuzumab	2382	149	0.78 (0.63–0.96)	0.02
OS				
Statin use				
No	4348	234	1.00	
Yes	417	34	1.16 (0.78–1.73)	0.45
Age group				
< 65 years	4161	214	1.00	
≥ 65 years	604	54	1.47 (1.05–2.06)	0.02
Menopausal status				
Premenopausal	2311	97	1.00	
Postmenopausal	2454	171	1.52 (1.15–2.01)	0.004
Hormone receptor status				
ER and/or PR positive	3148	147	1.00	
ER and PR negative	1617	121	1.46 (1.14–1.86)	0.002
BMI				
< 30 kg/m^2^	3911	213	1.00	
≥ 30 kg/m^2^ < 35 kg/m^2^	563	33	0.95 (0.65–1.38)	0.80
≥ 35 kg/m^2^	291	22	1.20 (0.76–1.89)	0.44
Diabetes				
No	4493	248	1.00	
Yes	272	20	0.94 (0.58–1.52)	0.80
Hypertension				
No	3700	189	1.00	
Yes	1065	79	1.11 (0.82–1.51)	0.49
Coronary heart disease				
No	4724	263	1.00	
Yes	41	5	1.78 (0.72–4.37)	0.21
Nodal status				
0 nodes	1785	48	1.00	
1–3 nodes	1795	85	1.84 (1.29–2.63)	< 0.001
≥ 4 nodes	1185	135	4.10 (2.91–5.78)	< 0.001
Tumor size				
0–1.9 cm	1906	72	1.00	
≥ 2 cm < 5 cm	2541	152	1.37 (1.03–1.82)	0.03
≥ 5 cm	318	44	2.40 (1.62–3.55)	< 0.001
Treatment arm				
Placebo	2383	143	1.00	
Pertuzumab	2382	125	0.87 (0.69–1.11)	0.27

None of the two-way interaction terms with statins use reached statistical significance. Therefore, the multivariate analysis reports only the main effects. For the multivariate analysis, only patients for whom all variables were available (*n* = 4765) were included

*BMI* body mass index, *CI* confidence interval, *DRFI* distant relapse-free interval, *ER* estrogen receptor, *IDFS* invasive disease-free survival; nodal status, number of positive lymph nodes, *OS* overall survival; *PR* progesterone receptor

**Table 3:** Multivariate analysis of IDFS, DRFI, and OS—Sensitivity analyses. No significant interaction between statin use, obesity, treatment arm, hormone receptor status, and survival could be detected (all *p*-values > 0.05). In multivariate analysis, factors that were associated with worse OS were larger tumor size, nodal positivity, hormone receptor negativity, older age and postmenopausal status. Factors associated with worse IDFS and DRFI were larger tumor size, nodal positivity and placebo treatment (Table 2). In a competing risk analysis with the outcome of interest being death due to breast cancer, statin treatment was not associated with a better or worse outcome (HR, 1.29; 95% CI, 0.77–2.19; *p*-value = 0.34)

Parameter	Patients, *n*	Events, *n*	Hazard ratio (95% CI)	*p*-value
Landmark at 12 months				
IDFS				
Statin use				
No	4160	399	1.00	
Yes	393	44	1.13 (0.80–1.59)	0.49
DRFI				
Statin use				
No	4160	276	1.00	
Yes	393	31	1.31 (0.86–1.97)	0.20
OS				
Statin use				
No	4160	198	1.00	
Yes	393	28	1.15 (0.74–1.78)	0.53
Start statin as time-dependent covariate				
IDFS				
Statin use				
No	4348	452	1.00	
Yes	417	51	1.11 (0.81–1.53)	0.52
DRFI				
Statin use				
No	4348	309	1.00	
Yes	417	32	1.21 (0.81–1.81)	0.35
OS				
Statin use				
No	4348	234	1.00	
Yes	417	34	1.17 (0.79–1.73)	0.44

*CI* confidence interval, *DRFI* distant relapse-free interval, *IDFS* invasive disease-free survival, *OS* overall survival

## Discussion

In this exploratory analysis of the APHINITY trial, we investigated the association of statin treatment and clinical outcomes of patients with early HER2 + BC treated with chemotherapy plus trastuzumab with or without pertuzumab. Previous studies provide clinical evidence suggesting a protective role of statins in patients with BC. In the BIG 1–98 trial, use of cholesterol-lowering medications was associated with improved outcome among 8010 postmenopausal patients with early-stage, hormone receptor-positive BC. Notably, initiation of treatment during endocrine therapy appeared to be associated with improved DFS (HR, 0.79; 95% CI, 0.66–0.95), breast cancer-free interval (HR, 0.76; 95% CI, 0.60–0.97) and distant recurrence-free interval (HR, 0.74; 95% CI, 0.56–0.97) [35]. A nationwide Danish prospective cohort study suggested that lipophilic statin use reduced the risk of recurrence at 10 years among 18,769 women with early-stage BC (HR, 0.73; 95% CI, 0.60–0.89). The protective effect was most pronounced in patients with hormone receptor-positive disease. The study included patients followed on the Danish Breast Cancer Cooperative Group registry between 1996 and 2003 [32]. Other studies were unable to show a protective correlation between statin use and BC recurrence. The Life After Cancer Epidemiology Study including 1945 early-stage BC patients (diagnosed between 1997 and 2000) failed to show a statistically significant reduction in BC recurrences in patients receiving statins (rate ratio, 0.67; 95% CI, 0.39–1.13). Of note, lipophilic statins were mainly prescribed [36]. In line with these data, an analysis from the German MARIEplus study of more than 3000 patients with BC did not provide clear supportive evidence for an association between lipid-lowering drugs and BC outcome – both in terms of recurrence and BC-specific mortality [27]. A post-hoc analysis of the ABCSG-18 trial including patients with hormone receptor-positive BC suggested a worse DFS (HR, 1.35; 95% CI, 1.04–1.75) of patients receiving concomitant statins. Yet, after correction for possible confounders (age, smoking status, adjuvant chemotherapy) this effect subsided [39].

Regarding death from BC, a recent cancer registry analysis of 14,976 women diagnosed with BC between 2007 and 2016 in New Zealand found a protective association between statin use and BC-specific death (HR, 0.74; 95% CI, 0.63–0.86). In subgroup analysis, this association was restricted to women with hormone receptor-positive BC, postmenopausal women, women with advanced stage disease and prevalent statin users [20]. A cohort study of 13,378 females diagnosed with BC between 1995 and 2013 in Finland reported a protective association between post-diagnostic statin use and death from BC when the median total cholesterol decreased subsequently (HR, 0.49; 95% CI, 0.32–0.75). Yet, this risk difference was only statistically significant in hormone receptor-positive BC and not evident in triple-negative or HER2 + BC [22]. A nationwide cohort study in Scotland of 15,140 newly diagnosed BC patients from 2009 to 2012 within the Scottish Cancer registry did not find clear evidence of a protective association between post-diagnostic statin use and BC-specific mortality (HR, 0.95; 95% CI, 0.79–1.15) [31]. A meta-analysis in 2016, which included 10 studies with BC patients, suggested that statin use was associated with improvement in recurrence-free survival, overall survival and cancer-specific survival. At least the improvement in recurrence-free survival appeared to be confined to the use of lipophilic statins [17]. A recent meta-analysis which included 23 studies showed that statin use was associated with lower BC recurrence, all-cause mortality and disease-specific mortality [18]. It cannot be ruled out that the protective association between statin use and overall survival observed in some studies is due to a cardioprotective role of statins in patients with hormone receptor-positive disease taking aromatase inhibitors. It is not clear if the duration of statin exposure might play a role with regard to survival outcomes of BC patients. In the previously mentioned cohort study in New Zealand the risk for BC death generally decreased with increasing statin dosage over time. Other studies suggest that statins become more protective with increasing dose [9, 10, 13, 16, 22], while others have found no evidence of a dose dependency [11, 15, 19, 23, 31, 36]. Furthermore, two clinical trials that examined statin treatment in the neoadjuvant setting suggested a positive effect on reducing tumor proliferation even when taking statins only for a very short time [44, 45].

The majority of studies in the literature have not reported results of statin use in HER2 + BC or detailed information for this subgroup is missing. Preclinical models suggest that the mevalonate pathway, which can be targeted with statins, acts as an escape mechanism of survival and growth in HER2 + BC resistant to anti-HER2 therapies [40]. In line with the subgroup analysis in the BIG 1–98 trial, we found that statin users were more likely to be diagnosed with smaller sized tumors than non-statin users [35]. Numerically there were slightly more distant relapses and more IDFS events among statin users than among non-statin users (7.8% vs 7.1% and 12.8% vs 10.4%, respectively) despite the fact that statin users were diagnosed with smaller sized tumors. Yet, in multivariate analysis (adjusted for tumor size, nodal status and the existence of comorbidities among others) and despite the biological rationale, the current analysis showed no association of statin use on IDFS, DRFI, or OS in this large patient cohort of early HER2 + BC. Hormone receptor status had no impact on the results. Statin use overall was associated with a trend to worse OS in univariate analysis. The results were not statistically significant and may be subject to residual confounding. It should come as no surprise that patients who take statins under the assumption of a concomitant disease could have a higher risk of death during the course of a clinical trial follow-up. In a competing risk analysis with the event of interest being death due to BC, statin treatment was not associated with outcome. Of note, statin users were treated less frequently with anthracycline-containing regimens than non-statin users which might have influenced the results. In contrast to some observational studies, neither lipophilic nor hydrophilic statin use was associated with an improved outcome.

### Study limitations

Some limitations exist in our study: (a) the exploratory nature of the analysis—the association between statin use and BC outcome was not a predefined hypothesis; (b) the vast majority of patients who received statins (91%) were receiving them at enrolment to APHINITY, although in 54% of cases the exact start date of statin co-medication was not available; (c) statin adherence was not systematically assessed and checked. Hence, we cannot exclude that some patients were non-adherent to treatment or might have received statins only for a short period of time; (d) the documentation of causes of death within APHINITY (especially considering competing events) might not have been optimal; e) lipid parameters were not collected either at baseline or during the course of the study, so it is not possible to draw any conclusions about a possible association with the outcome.

The strength of our analysis lies in the evaluation of a large, prospective phase 3 study with comprehensive information on patient and tumor characteristics and information on disease recurrence, type of recurrence, and deaths. To the best of our knowledge, our analysis is the largest focusing on statin co-medication in patients with early HER2 + BC.

In conclusion, in our analysis the use of statins within the APHINITY trial was not associated with improved outcomes in terms of IDFS, DRFI, and OS in patients with early HER2 + BC. Only a prospective, randomized study would be able to clarify whether the prognosis of patients with early breast cancer could be improved by statins. However, considering the high costs and complexity of running such a trial, it remains questionable whether this will ever happen. So far, apart from the current known indications for statin use, adding statins for early HER2-positive breast cancer is not recommended.

## Supplementary Information

Below is the link to the electronic supplementary material.

Supplementary file1 (DOCX 33 KB)

## Data Availability

Data are available upon reasonable request. Data and results are available at the Data Centre at Institut Jules Bordet in Brussels (Belgium) and can be made available upon approval of a research proposal.
