# Supplementary material for: Association of statin use on survival outcomes of patients with early-stage HER2-positive breast cancer in the APHINITY trial
Source: Breast Cancer Res Treat. 2025 Apr 28;212(1):57–69. doi: 10.1007/s10549-025-07699-2 (PMC12086115; doi:10.1007/s10549-025-07699-2)
Supplement: Supplementary file 1 — Supplementary file1 (DOCX 33 KB) [file 10549_2025_7699_MOESM1_ESM.docx]

**Association of statin use on survival outcomes of patients with early-stage HER2-positive breast cancer in the APHINITY trial**

**Authors**

Christian Maurer^1,2^; Elisa Agostinetto^3^; Lieveke Ameye^3^; Matteo Lambertini^4,5^; Samuel Martel^6,7^; Noam Ponde^8^; Mariana Brandão^3^; Francesca Poggio^5^; Arlindo Ferreira^9^; Rachel Schiff^10^; Carmine De Angelis^11^; Richard D. Gelber^12^; Susan Dent^13^; Christoph Thomssen^14^; Martine Piccart^3^; Evandro de Azambuja^3^

**Affiliations**

1 National Center for Tumor Diseases (NCT) Heidelberg, University Hospital and German Cancer Research Center Heidelberg, Im Neuenheimer Feld 460, 69120, Heidelberg, Germany

2 Department I of Internal Medicine, Center for Integrated Oncology Aachen Bonn Cologne Düsseldorf, Faculty of Medicine and University Hospital Cologne, University of Cologne, Cologne, Germany

3 Université Libre de Bruxelles (ULB), Hôpital Universitaire de Bruxelles (HUB), Institut Jules Bordet, Rue Meylemeersch 90, 1070 Bruxelles, Belgium

4 Department of Internal medicine and Medical Specialties (DiMI), School of Medicine, University of Genova, Genova, Italy

5 Department of Medical Oncology, U.O. Clinical di Oncologia Medica, IRCCS Ospedale Policlinico San Martino, Genova, Italy

6 Specialised Medicine Department, CISSS Montérégie-Centre/Hôpital Charles-Le Moyne, Greenfield Park, Québec, Canada

7 Université of Sherbrooke, Sherbrooke, Québec, Canada

8 Daiichi Sankyo, Clinical Development Department, Morristown, NJ, USA

9 Católica Medical School, Universidade Católica Portuguesa, Lisbon, Portugal

10 Lester and Sue Smith Breast Center, Departments of Medicine and of Molecular and Cellular Biology, Baylor College of Medicine, Houston, TX, USA

11 Medical Oncology Unit, Department of Clinical Medicine and Surgery, University of Naples “Federico II”, Naples, Italy

12 Harvard Medical School, Harvard TH Chan School of Public Health, Dana-Farber Cancer Institute, Frontier Science Foundation, Boston, MA, USA

13 Wilmot Cancer Institute, Department of Medicine, University of Rochester, Rochester NY, USA

14 Department of Gynaecology, Martin-Luther-University Halle-Wittenberg, Halle (Saale), Germany

**Correspondence to**

Christian Maurer
National Center for Tumor Diseases (NCT) Heidelberg, University Hospital and German Cancer Research Center Heidelberg, Heidelberg, Germany
Im Neuenheimer Feld 460, 69120 Heidelberg, Germany
Tel: +49 6221-56 36658
Fax: +49 6221-56 5614
ORCID 0000-0003-4388-5163
E-mail: Christian.Maurer@med.uni-heidelberg.de

**Supplementary Table 1:** Univariate analysis of IDFS, DRFI, and OS

| **Parameter** | **Patients, n** | **Events, n** |  | **Hazard ratio (95% CI)** | ***p*-value** |
| --- | --- | --- | --- | --- | --- |
| **IDFS** | | | | | |
| All | 4,804 | 508 | Statin (yes vs no) | 1.27 (0.95-1.68) | 0.10 |
|  |  |  | Lipophilic statin (yes vs no)^a^ | 1.28 (0.93-1.77) | 0.14 |
|  |  |  | Hydrophilic statin (yes vs no) | 1.14 (0.67-1.94) | 0.63 |
| Pertuzumab arm | 2,400 | 221 | Statin (yes vs no) | 1.58 (1.06-2.37) | 0.03 |
|  |  |  | Lipophilic statin (yes vs no)^a^ | 1.76 (1.13-2.73) | 0.01 |
|  |  |  | Hydrophilic statin (yes vs no) | 1.01 (0.41-2.44) | 0.99 |
| Placebo arm | 2,404 | 287 | Statin (yes vs no) | 1.04 (0.70-1.54) | 0.85 |
|  |  |  | Lipophilic statin (yes vs no)^a^ | 0.95 (0.59-1.52) | 0.82 |
|  |  |  | Hydrophilic statin (yes vs no) | 1.21 (0.62-2.35) | 0.58 |
| Premenopasusal | 2,325 | 245 | Statin (yes vs no) | 1.40 (0.58-3.40) | 0.45 |
|  |  |  | Lipophilic statin (yes vs no)^a^ | 1.92 (0.71-5.15) | 0.20 |
|  |  |  | Hydrophilic statin (yes vs no) | 0.67 (0.09-4.78) | 0.69 |
| Postmenopausal | 2,462 | 260 | Statin (yes vs no) | 1.19 (0.87-1.64) | 0.28 |
|  |  |  | Lipophilic statin (yes vs no)^a^ | 1.20 (0.84-1.72) | 0.31 |
|  |  |  | Hydrophilic statin (yes vs no) | 1.05 (0.57-1.92) | 0.88 |
| Hormone receptor-negative | 1,632 | 188 | Statin (yes vs no) | 1.29 (0.81-2.05) | 0.28 |
|  |  |  | Lipophilic statin (yes vs no)^a^ | 1.39 (0.82-2.36) | 0.22 |
|  |  |  | Hydrophilic statin (yes vs no) | 1.00 (0.41-2.44) | 1.00 |
| Hormone receptor-positive | 3,172 | 320 | Statin (yes vs no) | 1.25 (0.88-1.78) | 0.22 |
|  |  |  | Lipophilic statin (yes vs no)^a^ | 1.22 (0.81-1.84) | 0.34 |
|  |  |  | Hydrophilic statin (yes vs no) | 1.22 (0.63-2.36) | 0.56 |
| BMI 20.0-24.9 kg/m^2^ | 2,425 | 235 | Statin (yes vs no) | 1.01 (0.58-1.77) | 0.97 |
|  |  |  | Lipophilic statin (yes vs no)^a^ | 1.08 (0.58-2.04) | 0.81 |
|  |  |  | Hydrophilic statin (yes vs no) | 0.79 (0.25-2.45) | 0.68 |
| BMI 25.0-29.9 kg/m^2^ | 1,392 | 163 | Statin (yes vs no) | 1.39 (0.88-2.17) | 0.15 |
|  |  |  | Lipophilic statin (yes vs no)^a^ | 1.52 (0.93-2.48) | 0.09 |
|  |  |  | Hydrophilic statin (yes vs no) | 0.90 (0.33-2.41) | 0.83 |
| BMI ≥ 30 kg/m^2^ | 860 | 101 | Statin (yes vs no) | 1.21 (0.74-2.00) | 0.45 |
|  |  |  | Lipophilic statin (yes vs no)^a^ | 1.01 (0.55-1.85) | 0.97 |
|  |  |  | Hydrophilic statin (yes vs no) | 1.63 (0.76-3.52) | 0.21 |
| **DRFI** | | | | | |
| All | 4,804 | 343 | Statin (yes vs no) | 1.13 (0.79-1.61) | 0.52 |
|  |  |  | Lipophilic statin (yes vs no)^a^ | 1.22 (0.82-1.82) | 0.33 |
|  |  |  | Hydrophilic statin (yes vs no) | 0.83 (0.39-1.75) | 0.62 |
| Pertuzumab arm | 2,400 | 149 | Statin (yes vs no) | 1.35 (0.80-2.26) | 0.26 |
|  |  |  | Lipophilic statin (yes vs no)^a^ | 1.76 (1.03-2.99) | 0.04 |
|  |  |  | Hydrophilic statin (yes vs no) | 0.29 (0.04-2.04) | 0.21 |
| Placebo arm | 2,404 | 194 | Statin (yes vs no) | 0.96 (0.59-1.59) | 0.88 |
|  |  |  | Lipophilic statin (yes vs no)^a^ | 0.85 (0.46-1.57) | 0.61 |
|  |  |  | Hydrophilic statin (yes vs no) | 1.20 (0.53-2.71) | 0.66 |
| Premenopasusal | 2,325 | 180 | Statin (yes vs no) | 1.11 (0.35-3.46) | 0.86 |
|  |  |  | Lipophilic statin (yes vs no)^a^ | 1.84 (0.59-5.76) | 0.29 |
|  |  |  | Hydrophilic statin (yes vs no) | Not evaluable |  |
| Postmenopausal | 2,462 | 162 | Statin (yes vs no) | 1.20 (0.80-1.79) | 0.37 |
|  |  |  | Lipophilic statin (yes vs no)^a^ | 1.28 (0.82-1.99) | 0.28 |
|  |  |  | Hydrophilic statin (yes vs no) | 0.90 (0.40-2.03) | 0.79 |
| Hormone receptor-negative | 1,632 | 125 | Statin (yes vs no) | 1.04 (0.56-1.92) | 0.91 |
|  |  |  | Lipophilic statin (yes vs no)^a^ | 1.24 (0.63-2.44) | 0.53 |
|  |  |  | Hydrophilic statin (yes vs no) | 0.58 (0.14-2.36) | 0.45 |
| Hormone receptor-positive | 3,172 | 218 | Statin (yes vs no) | 1.18 (0.76-1.83) | 0.47 |
|  |  |  | Lipophilic statin (yes vs no)^a^ | 1.21 (0.74-1.99) | 0.44 |
|  |  |  | Hydrophilic statin (yes vs no) | 0.99 (0.41-2.40) | 0.98 |
| BMI 20.0-24.9 kg/m^2^ | 2,425 | 156 | Statin (yes vs no) | 0.82 (0.38-1.74) | 0.60 |
|  |  |  | Lipophilic statin (yes vs no)^a^ | 1.15 (0.54-2.45) | 0.72 |
|  |  |  | Hydrophilic statin (yes vs no) | Not evaluable |  |
| BMI 25.0-29.9 kg/m^2^ | 1,392 | 116 | Statin (yes vs no) | 1.12 (0.63-1.99) | 0.70 |
|  |  |  | Lipophilic statin (yes vs no)^a^ | 1.15 (0.60-2.20) | 0.67 |
|  |  |  | Hydrophilic statin (yes vs no) | 0.95 (0.30-3.00) | 0.94 |
| BMI ≥ 30 kg/m^2^ | 860 | 64 | Statin (yes vs no) | 1.30 (0.70-2.38) | 0.41 |
|  |  |  | Lipophilic statin (yes vs no)^a^ | 1.21 (0.60-2.45) | 0.60 |
|  |  |  | Hydrophilic statin (yes vs no) | 1.38 (0.50-3.80) | 0.53 |
| **OS** | | | | | |
| All | 4,804 | 272 | Statin (yes vs no) | 1.62 (1.14-2.31) | 0.007 |
|  |  |  | Lipophilic statin (yes vs no)^a^ | 1.58 (1.05-2.36) | 0.03 |
|  |  |  | Hydrophilic statin (yes vs no) | 1.55 (0.82-2.91) | 0.18 |
| Pertuzumab arm | 2,400 | 125 | Statin (yes vs no) | 1.89 (1.15-3.12) | 0.01 |
|  |  |  | Lipophilic statin (yes vs no)^a^ | 1.98 (1.14-3.46) | 0.02 |
|  |  |  | Hydrophilic statin (yes vs no) | 1.42 (0.52-3.84) | 0.49 |
| Placebo arm | 2,404 | 147 | Statin (yes vs no) | 1.41 (0.86-2.30) | 0.18 |
|  |  |  | Lipophilic statin (yes vs no) ^a^ | 1.26 (0.70-2.27) | 0.45 |
|  |  |  | Hydrophilic statin (yes vs no) | 1.64 (0.73-3.72) | 0.23 |
| Premenopasusal | 2,325 | 98 | Statin (yes vs no) | 2.79 (1.03-7.60) | 0.04 |
|  |  |  | Lipophilic statin (yes vs no)^a^ | 3.44 (1.09-10.86) | 0.04 |
|  |  |  | Hydrophilic statin (yes vs no) | 1.74 (0.24-12.45) | 0.58 |
| Postmenopausal | 2,462 | 172 | Statin (yes vs no) | 1.16 (0.79-1.73) | 0.45 |
|  |  |  | Lipophilic statin (yes vs no)^a^ | 1.19 (0.77-1.85) | 0.44 |
|  |  |  | Hydrophilic statin (yes vs no) | 1.00 (0.47-2.13) | 1.00 |
| Hormone receptor-negative | 1,632 | 122 | Statin (yes vs no) | 1.53 (0.89-2.62) | 0.12 |
|  |  |  | Lipophilic statin (yes vs no)^a^ | 1.93 (1.09-3.44) | 0.02 |
|  |  |  | Hydrophilic statin (yes vs no) | 0.61 (0.15-2.47) | 0.49 |
| Hormone receptor-positive | 3,172 | 150 | Statin (yes vs no) | 1.71 (1.08-2.71) | 0.02 |
|  |  |  | Lipophilic statin (yes vs no)^a^ | 1.36 (0.77-2.39) | 0.30 |
|  |  |  | Hydrophilic statin (yes vs no) | 2.43 (1.19-4.94) | 0.01 |
| BMI 20.0-24.9 kg/m^2^ | 2,425 | 115 | Statin (yes vs no) | 1.30 (0.63-2.66) | 0.48 |
|  |  |  | Lipophilic statin (yes vs no)^a^ | 1.35 (0.59-3.07) | 0.47 |
|  |  |  | Hydrophilic statin (yes vs no) | 1.08 (0.27-4.35) | 0.92 |
| BMI 25.0-29.9 kg/m^2^ | 1,392 | 98 | Statin (yes vs no) | 2.02 (1.21-3.37) | 0.007 |
|  |  |  | Lipophilic statin (yes vs no)^a^ | 2.21 (1.27-3.82) | 0.005 |
|  |  |  | Hydrophilic statin (yes vs no) | 1.18 (0.37-3.72) | 0.78 |
| BMI ≥ 30 kg/m^2^ | 860 | 56 | Statin (yes vs no) | 1.11 (0.56-2.20) | 0.76 |
|  |  |  | Lipophilic statin (yes vs no)^a^ | 0.73 (0.29-1.82) | 0.50 |
|  |  |  | Hydrophilic statin (yes vs no) | 2.08 (0.83-5.21) | 0.12 |

**Abbreviations:**

BMI, body mass index; CI, confidence interval; DRFI, distant relapse-free interval; IDFS, invasive-disease-free survival; OS, overall survival.

a This group includes also patients with both lipophilic and hydrophilic statin use (n=5).
